# ABC Transport Proteins in Cardiovascular Disease—A Brief Summary

**DOI:** 10.3390/molecules22040589

**Published:** 2017-04-06

**Authors:** Toni Schumacher, Ralf A. Benndorf

**Affiliations:** Institute of Pharmacy, Department of Clinical Pharmacy and Pharmacotherapy, Martin-Luther-University Halle-Wittenberg, Wolfgang-Langenbeck-Strasse 4, D-06120 Halle (Saale), Germany; toni.schumacher@med.uni-rostock.de

**Keywords:** ABC transporter, cardiovascular diseases, atherosclerosis, pharmacokinetic interactions, pharmacogenetics

## Abstract

Adenosine triphosphate (ATP)-binding cassette (ABC) transporters may play an important role in the pathogenesis of atherosclerotic vascular diseases due to their involvement in cholesterol homeostasis, blood pressure regulation, endothelial function, vascular inflammation, as well as platelet production and aggregation. In this regard, ABC transporters, such as ABCA1, ABCG5 and ABCG8, were initially found to be responsible for genetically-inherited syndromes like Tangier diseases and sitosterolemia. These findings led to the understanding of those transporter’s function in cellular cholesterol efflux and thereby also linked them to atherosclerosis and cardiovascular diseases (CVD). Subsequently, further ABC transporters, i.e., ABCG1, ABCG4, ABCB6, ABCC1, ABCC6 or ABCC9, have been shown to directly or indirectly affect cellular cholesterol efflux, the inflammatory response in macrophages, megakaryocyte proliferation and thrombus formation, as well as vascular function and blood pressure, and may thereby contribute to the pathogenesis of CVD and its complications. Furthermore, ABC transporters, such as ABCB1, ABCC2 or ABCG2, may affect the safety and efficacy of several drug classes currently in use for CVD treatment. This review will give a brief overview of ABC transporters involved in the process of atherogenesis and CVD pathology. It also aims to briefly summarize the role of ABC transporters in the pharmacokinetics and disposition of drugs frequently used to treat CVD and CVD-related complications.

## 1. Introduction

Key functions, such as nutrient intake, sequestration of metabolic end products at the most primary level and the exchange of compounds between cellular organelles or even tissues in more advanced life forms, often take place against concentration gradients across cellular membranes. It is therefore not surprising that in simple life forms like bacteria, almost ten percent of the entire genome is dedicated to proteins that are involved in transport processes in the form of membrane-bound or soluble proteins [1]. Such transport processes against chemical gradients always require the use of free energy either by simultaneous use of an opposing electrochemical potential difference (secondary active transport) or a coupled enzymatic reaction exploiting the chemical energy of Adenosine triphosphate (ATP) hydrolysis (primary active transport). These ATP-driven transport proteins are called ATPases and compose a large superfamily including F-, A- and V-ATPases, P-type ATPases and ABC (ATP-binding cassette) transporters. While the other ATPases are predominantly motor enzymes or transporters with a very limited substrate range, ABC transporters generally cover a wide spectrum of different substrates from small inorganic and organic molecules to larger organic compounds. The transport direction is thereby always of a unidirectional nature and, apart from a few exceptions in prokaryotic cells, limited to export functions. With the introduction of differentiated tissues and organs during the evolution of eumetazoa, transport processes became even more crucial since now substrates, metabolites, metabolic end products and signaling molecules not only had to be exchanged between cellular organelles or different cells at the most, but also between different organs depending on the exchange of different organic and inorganic compounds. Unsurprisingly, ABC transporters are present in almost all prokaryotes and virtually all cell types of eukaryotes. Consequently, ABC transporters are most abundantly expressed in organs with high metabolic rates and in endothelial tissues isolating certain organs from the rest of the body like the blood-brain barrier, blood-testis barrier and others.

### 1.1. Functional Properties of ABC Transporters

Due to their functional and architectural characteristics, ABC transporters are divided into three subclasses including two groups of importers and one group of exporters. While ABC importers are predominantly found in prokaryotes where they manage the nutrient and ion intake [2,3], they only sparsely occur in eukaryotes. The vast majority of ABC transporters expressed in eukaryotes are ABC exporters facilitating export functions such as secreting dietary agents, exporting metabolites or even transport signaling molecules. Particular members of this group are also involved in nuclear processes like DNA repair, chromatin reorganization [4], telomere maintenance [5] and RNA trafficking, as well [6].

There are currently 49 different genes known to be encoding ABC transporters in humans. These transporters are involved in a broad range of cellular processes, therein transporting a large number of different hydrophobic compounds across cellular boundaries. Since several of those genes are alternatively spliced during transcription, each of those 49 genes not only encode one single protein, but instead often a multiple of different ABC protein variants [7]. According to the cytochrome P450 (CYP) enzyme gene nomenclature system, all of the ABC genes are divided into seven subfamilies named ABCA–ABCG based mainly on similarity in gene structure, e.g., half vs. full transporters, and on sequence homology in the nucleotide-binding domains (NBDs) and transmembrane domains (TMDs) [8].

Still, all ABC transporters share a common structure that is characterized by being composed of two TMDs and two cytosolic ATP-binding domains also known as NBDs. While in prokaryotes, the four TMD and NBD subunits are expressed as individual polypeptides, they are fused to a single protein in eukaryotes. Differing from the standard eukaryotic “full transporter” configuration, comprised of all four subunits, also dimeric “half transporter” types are known, which consist of either two identical (homodimeric) or different (heterodimeric) halves. The multiple hydrophobic TMD segments comprised of 6–10 transmembrane spanning α-helices anchor the protein within the lipid bilayer of the cellular membrane. These 12–20 transmembrane spanning domains of a full transporter thereby line the transmembrane pore and by their specific residues determine the transporter’s substrate specificity. Most ABC transporters are capable of transporting a broad spectrum of substrates. In P-glycoprotein (ABCB1), for example, different overlapping drug binding sites have been identified within the transmembrane channel enabling this polyspecificity [9]. Unsurprisingly, the TMD sequence variations are numerous since they determine the transporter’s substrate range. In contrast, the NBD substructures of eukaryotic ABC transporters, necessary to utilize the free energy from ATP-binding and hydrolysis for substrate transport, are highly conserved between species [8,10]. The signature sequence Leu-Ser-Gly-Gly-Gln (LSGGQ) within the NBD’s α-helical domain and the Walker A motifs are common characteristics of these substructures and form the NBD’s active center. Thereby, two ATP molecules are bound between the phosphate-binding loop (P-loop) residue of NBD1 and the signature domain of NBD2 and vice versa. This tight coupling of ATP molecules within the NBDs is necessary for ATP hydrolysis and utilization of its chemical energy, whereas the precise mechanism of ATP binding, hydrolysis and energy conversion is still controversial [11]. Nonetheless, most data support a model in which ATP binding and hydrolysis are coupled to conformational changes (open vs. closed conformation) in the ABC transporter molecule to support substrate extrusion. In this model, the ABC transporter initially shows an open dimer configuration with low affinity of NBDs towards ATP and an open (high affinity) substrate binding pocket. Upon binding of a substrate to its TMD target region, conformational changes within the transporter molecule increase the NBD’s ATP affinity. The binding of two ATP molecules then changes the protein conformation to a closed dimer configuration. The ATP-driven change between open and closed dimer conformation also induces conformational changes in the TMD, resulting in substrate translocation. The cycle ends with ATP hydrolysis and reconfiguration of the whole protein to its initial resting state after consecutively releasing phosphate (P) and adenosine diphosphate (ADP).

### 1.2. ABC Transporters in the Pathogenesis of Cardiovascular Disease 

With more than 17.3 million deaths per year, cardiovascular diseases (CVDs) remain the greatest cause of deaths worldwide, a number that is predicted to grow to more than 23 million by 2030. In this context, one has to additionally keep in mind that CVDs are causing more than half of all premature deaths in Europeans aged 65–74 years, with nearly half of those people dying from coronary artery or ischemic heart disease and almost a third from cerebrovascular diseases [12]. Although the term “cardiovascular disease” includes all diseases that involve the heart and blood vessels regardless of their pathogenesis, the vast majority of CVDs are caused by atherosclerotic vascular disease or its complications [13]. Atherosclerosis is a chronic, immunoinflammatory disease of the vascular wall that is heavily influenced by well-characterized risk factors, such as hypercholesterolemia, hypertension, diabetes, smoking, male gender and age.

The process of atherosclerotic plaque formation is initiated by endothelial dysfunction, which facilitates subendothelial accumulation of low density lipoproteins (LDL) into the extracellular matrix of the blood vessels’ intima. Binding of LDL to negatively-charged extracellular matrix components causes retention of the LDL particles in the intima, where the LDL particles become susceptible to oxidative modification, e.g., by reactive oxygen species released from inflammatory cells [14]. Oxidized forms of LDL (OxLDL) not only are cytotoxic in higher concentrations, but also promote vascular inflammation by further activating endothelial cells and subsequently inducing intimal immune cell infiltration and formation of macrophage-like lipid-loaded foam cells [15,16]. Subsequent leukocyte recruitment further increases the plaque volume [16]. Progression of these “fatty streak“ lesions involves plaque growth, development and enlargement of a necrotic plaque core, as well as fibroatheromatous plaque remodeling through proliferation and migration of vascular smooth muscle cells. These processes drive the formation of complex unstable lesions that favor the occurrence of acute thrombotic complications. The expanding necrotic core tends to destabilize the plaque and can induce plaque rupture, which in turn leads to thrombus formation due to the release of vast amounts of matrix molecules that induce platelet activation. Indeed, acute clinical events, such as unstable angina, acute myocardial infarction, sudden coronary death or stroke, are hallmarks of clinically-manifest atherosclerosis and strongly affect the prognosis of patients suffering from CVD.

With at least 20 out of 49 human ABC transporters being related to the transport of lipids or lipid-like compounds within the human organism, it is not surprising that some of these transporters have been already linked to the pathogenesis of atherosclerotic vascular diseases. Moreover, ABC transporters have also been associated with vascular endothelial homeostasis and blood pressure regulation, as well as platelet production and aggregation and may thereby affect the course of CVD. Thus, the objective of this review is to summarize briefly current findings on the role of ABC transporters involved in the process of atherogenesis and CVD pathology and to outline the role of ABC transporters in the pharmacokinetics and disposition of drugs frequently used to treat CVD and CVD-related complications.

## 2. ABC Transporters Involved in Lipid Homeostasis and Trafficking

A hallmark of atherosclerotic vascular disease is the accumulation of lipids and cholesterol in the blood vessel’s sub-endothelial space and intimal foam cells. Several ABC transporters have been identified to play a major role in cellular efflux and trafficking of lipids and cholesterol. Thus, impairment of certain ABC transporters leads to enhanced atherogenesis. The other way around, targeted activation of these mediators might bring up new treatment options against atherosclerosis-derived CVD.

### 2.1. ABCA1

ABCA1 is probably the most prominent member of the ABC superfamily when it comes to atherosclerosis pathogenesis since it is crucial for high density lipoprotein (HDL) formation and, alongside other transporters, also for loading HDL with phospholipids and cholesterol. HDL is mostly formed within the liver [17] and to a lesser extent in the intestine [18] by the transfer of cholesterol and phospholipids apolipoprotein A-1 (ApoA-1) with ABCA1 being mainly responsible for this step. ABCA1 has thereby not only catalyzing functions in HDL formation. It also works as a cellular efflux transporter of cholesterol and lipids in vascular endothelial cells and macrophages. Hence, dysfunction of ABCA1 will significantly decrease serum HDL levels and thereby dramatically impair cholesterol and lipid transport functions. A known, but very rare autosomal recessive disorder caused by the lack of functional ABCA1 protein is called Tangier’s disease [19]. Patients suffering from this disease show a virtual absence of serum HDL along with hypertriglyceridemia and a reduction in LDL serum levels. Defective cholesterol and lipid transport result in the accumulation of cholesteryl-esters in different reticuloendothelial cells of various tissues like tonsils, lymph nodes, bone marrow, thymus, spleen, liver and intestinal mucosa. Even smooth muscle cells, fibroblasts and neuronal Schwann cells show lipid depositions [20]. Not only that HDL has anti-oxidant, anti-inflammatory, vasodilating and antithrombotic properties, it also induces anti-atherosclerotic effects due to its role as a cholesterol and phospholipid transporter that allows macrophage-like foam cells, endothelial cells and smooth muscle cells to remove their oxysterol and phospholipid overload [21]. In line with these observations, patients suffering from Tangier’s disease have a six-fold higher incidence of coronary heart disease [22]. So far, about twenty different mutations in the *ABCA1* gene have been described, which are all associated with a Tangier’s disease-like phenotype [23]. The fact that approximately 10% of individuals with very low HDL serum levels have been reported to inherit certain ABCA1 variants highly emphasizes the importance of ABCA1 for HDL homeostasis [24,25]. According to the latest observations, this reverse cholesterol transport also seems to be relevant to hematopoietic stem cell homeostasis. In vitro studies have shown that HDL reduces the proliferative capacity of granulocyte/macrophage colony-forming cells in an ABC transporter-dependent manner. It was found that membranous cholesterol content is linked to interleukin 3 (IL3)/granulocyte-macrophage colony-stimulating factor (GM-CSF) receptor expression levels of these cells, a receptor that controls proliferative activity via rat sarcoma (Ras)/extracellular-signal regulated kinases (ERK) signaling. It is thought to be mechanistic that ABCA1 and ABCG1 efflux cholesterol to HDL, thereby decreasing membranous cholesterol content and subsequently diminishing surface expression of the IL3/GM-CSF receptor [26]. Consequently, loss of function of these transporters would result in increased hematopoietic stem cell proliferation leading to leukocytosis, a phenomenon already described in respective knockout mouse models [27].

### 2.2. ABCA5

Even though studies reported that ABCA5 is involved in cellular lipid efflux, the role of the transporter in atherosclerotic vascular disease is still elusive. It was found that ABCA5 is expressed in monocytes/macrophages, cardiomyocytes, oligodendrocytes and astroglia of the brain. Moreover, ABCA5 expression increased in monocytes/macrophages after incubation with acetylated LDL [28]. Animal experiments were conducted using *ABCA5* knockout mice and *ABCA5*^−/−^ bone marrow transplants in order to elucidate *ABCA5* function. Wild-type animal hosts revealed a significantly impaired ABCG1-independent macrophage cholesterol efflux to HDL after receiving *ABCA5*^−/−^ bone marrow transplants, whereas knockout animals showed lysosomal-disease-like symptoms with enlarged ventricles and dilated cardiomyopathy leading to the depression of the cardiovascular system and early premature death [29]. When ABCA5 was only disrupted in bone marrow-derived cells, macrophage cholesterol efflux to HDL was indeed impaired while cellular cholesterol efflux to ApoA-1 was instead increased. Loading of ABCA5-deficient macrophages with OxLDL also led to increased foam cell formation. Nonetheless, transplantation of ABCA5-deficient macrophages into LDL knockout mice increased atherosclerotic lesions only in female, but not in male mice [29]. Thus, whether or not ABCA5 plays a significant role in CVD pathogenesis is still rather unclear and needs further research.

### 2.3. ABCG1

Besides OxLDL, also oxysterols are found abundantly in plaque deposits [30] and have been described to affect vascular homeostasis by inducing the dysfunction and apoptosis of vascular endothelial cells [31], smooth muscle cells [32] and macrophages [33]. An efficient vascular oxysterol clearance may therefore represent a relevant mechanism to counteract atherogenesis. Apart from ABCA1 and its role as the key player in HDL formation, ABCG1 is a potent cholesterol and oxysterol transporter, able to transfer cholesterol from macrophages or vascular endothelial cells to mature HDL particles, which represent a large proportion of overall plasma HDL. The so-called reverse cholesterol transport pathway, driven by ABCG1, is therefore a critical cholesterol and lipid clearance mechanism responsible for the transfer of excess cholesterol from periphery tissues back to the liver [34,35]. Consequently, dysfunction of ABCG1 results in an impaired cellular cholesterol and lipid efflux, an effect that has been demonstrated in vitro, when ABCG1-transfected cells showed a selective 7-ketocholesterol export to HDL [34], and in vivo using ABCG1 loss- and gain-of-function approaches in mice [36]. Herein, significant accumulation of cholesterol, triglycerides and phospholipids were found in several organs of *ABCG1*^−/−^ mice when fed with a Western diet, while this effect was absent when *ABCG1*^−/−^ mice also transgenically expressed the human ABCG1 transporter [37]. Recently, even clinical observations have suggested a significant impact of ABCG1 on monocyte function in atherosclerotic processes when decreased ABCG1 messenger RNA (mRNA) levels were found to be inversely correlated with the severity of coronary artery stenosis in CVD patients [38]. According to these observations, the ABCG1-driven macrophage reverse cholesterol transport mechanism may be viewed as a crucial atheroprotective process by which excess cholesterol is transported to the liver and excreted into the bile [39]. As described previously for ABCA1, ABCG1 is comparably relevant for hematopoietic stem cell functions due to its involvement in membranous cholesterol efflux to HDL [26].

### 2.4. ABCG5/G8

In contrast to the previously-discussed ABC transporters, ABCG5 and ABCG8 have been more indirectly associated with atherogenic processes by altering plasma sterol concentrations. Both of the two genes *ABCG5* and *ABCG8* are located head to head on chromosome 2p21, and each gene encodes a “half-transporter” protein that is non-functional in the monomeric state [18]. However, assembly of an ABCG5/G8 heterodimer, driven by the adipocyte-derived hormone leptin, leads to the formation of the fully-functional transporter ABCG5/G8 [18]. Decades ago, the first report on a syndrome now known as sitosterolemia appeared [18], which later on was linked to mutations in the *ABCG5* and *ABCG8* genes leading to loss of function of the ABCG5/G8 transporter. This very rare autosomal recessive disorder is characterized by significantly elevated plasma levels of plant sterols due to excessive absorbance of dietary sitosterol from the intestine. Apart from other clinical manifestations, premature atherosclerosis was particularly observed to affect male patients at a young age leading to CVDs like angina pectoris and myocardial infarctions [18,40]. It has been shown later on that ABCG5/G8 is exclusively expressed on the apical membranes of enterocytes and hepatocytes [41]. It is responsible for the efflux of cholesterol and plant sterols either by hepatocytes into the bile or through the enterocytes of the intestine back into the intestinal lumen for fecal disposal. Animal loss- and gain-of-function studies showed either 2–3-fold increased fractional absorption of dietary cholesterol when ABCG5/G8 was knocked out [18] or, vice versa, a 50% decrease when ABCG5/G8 was overexpressed [42]. Overexpression of ABCG5/G8 in liver and intestine also lowered cholesterol levels and aortic atherosclerotic lesion area in low-density lipoprotein receptor (Ldlr)-deficient mice [18]. All of these animal studies give functional explanations for the genetic polymorphisms and their respective phenotypes already described in humans [43,44,45]. Those phenotypes range from decreased [43] to elevated cholesterol absorption [44] and can even be directly linked to increased risk for CVD development [45].

### 2.5. ABCB4

The transport protein ABCB4 is also responsible for certain aspects of hepatic cholesterol handling and pathogenesis of CVD. It was first found expressed in different tissues like liver, heart, muscle, spleen, adrenal gland and tonsils [46]. Animal studies on ABCB4 knockout mice later revealed a severe hepatic phenotype when animals showed significant damage to hepatocytes and the bile ducts of the liver [47]. The damage was a result of impaired phospholipid and cholesterol transport from the liver into the bile. Later on, it was discovered that loss of ABCB4 function is also responsible for a comparable syndrome in humans who develop a progressive familial cholestasis. Beside ABCB4 being heavily expressed in the canicular membrane of hepatocytes, where it is crucial for the hepatic phosphatidyl choline efflux, it was also found in monocytes and macrophages [48]. In these cells, ABCB4 expression is additionally regulated by cholesterol levels [49]. It is therefore thought that ABCB4 might play a role in macrophage lipid homeostasis. Animal studies using depletion and subsequent transplantation of *ABCB4*^−/−^ bone marrow cells into an atherosclerosis mouse model had opposing effects on disease parameters, yet in the end, aggravated atherosclerosis pathology. Interestingly, in this experimental model, transplantation of *ABCB4*^−/−^ bone marrow cells aggravated atherogenesis even though the same animals simultaneously showed significantly decreased serum cholesterol levels. As *ABCB4*-deficient macrophages showed an increase in scavenger receptor A (SR-A), scavenger receptor class B type I (SR-BI) and cluster of differentiation 36 (CD36), the authors speculated that *ABCB4* deficiency may increase the ability of tissue-resident macrophages, as well as macrophage-like foam cells to scavenge and deposit LDL in various tissues, as well as in atherosclerotic blood vessels; thereby reducing serum cholesterol levels while increasing atherosclerotic plaque formation [50]. This hypothesis might explain the elevated foam cell generation and increased atherosclerotic lesions found in *ABCB4*^−/−^ bone marrow transplanted mice.

## 3. ABC Transporters in Atherothrombotic Diseases

Endothelial dysfunction and formation of complex unstable lesions during the process of atherogenesis not only lead to changes in vascular tone and luminal narrowing of affected blood vessels, but also favor the occurrence of acute thrombotic complications like myocardial infarction or stroke. In the precipitation of acute events (but also in the process of atherogenesis), platelets are crucial effector cells when it comes to the decisive thrombotic events. Imbalances in platelet count, size or function can therefore aggravate the course of atherosclerotic vascular disease and the prognosis of CVD patients. ABC transporters like ABCB6, ABCC4 or ABCG4 have been implicated in platelet differentiation and function and may therefore be relevant for CVD pathogenesis.

### 3.1. ABCG4

ABCG4 shows high levels of homology to ABCG1. It is therefore thought to be involved in cholesterol efflux to HDL, as has been already observed in vitro [51]. Since it was not found expressed in foam cells, but rather in brain [52] and hematopoietic tissue, like fetal liver and bone marrow [52,53], its role in atherosclerosis progression remained uncertain. This expression pattern hints at a functional role of ABCG4 in certain stem cell subsets [54], even more so because membranous cholesterol homoeostasis and cellular sterol metabolism were long before found to be connected to proliferative functions [55] and have also already been linked to certain transport proteins like ABCG2 [56], a protein that is highly homologues to ABCG4. Concordant with these premises, animal studies showed alterations in bone marrow functions due to loss of ABCG4 expression. Using an in vivo approach in *ABCG4*^−/−^ mice, it was found that ABCG4 plays a critical role in cholesterol efflux from megakaryocyte progenitors (MkPs). ABCG4, due to its role as a cholesterol efflux transporter, acts as a cholesterol sensor in close coordination with Lck/Yes novel (LYN) kinase. As an upstream regulator of E3 ligase casitas B-lineage lymphoma (c-CBL), LYN kinase then activates c-CBL leading to internalization and ubiquitination of thrombopoietin receptor (c-MPL), which as a result completes the negative feedback regulation of cholesterol sensing on proliferative activities in MkPs [51].

Hematopoietic homeostasis was altered in the sense of accelerated megakaryocyte proliferation, which as a result led to thrombocytosis. These animals not only showed increased platelet counts, but also increased numbers of platelet-neutrophil and -monocyte aggregates, which expressed higher levels of CD11b and thereby indicate a higher state of activation. As a result, atherosclerotic lesions were significantly more severe in atherosclerosis-prone *ABCG4*^-/-^ mice when fed with a Western diet containing high amounts of fat and cholesterol [51]. Hence, ABCG4 appears to influence platelet maturation and thereby aggravate atherogenesis and thrombotic complications.

### 3.2. ABCB6

Shortly after the finding of ABCG4 being crucially involved in hematopoietic/megakaryocyte proliferation and differentiation, experimental studies on ABC knockout mice revealed another ABC transporter involved in megakaryocyte functions. Animals, deficient for ABCB6, also showed very comparable phenotypes to those lacking ABCG4. Megakaryopoiesis was also enhanced, leading to increased platelet counts, platelet volume and even to increased platelet activity. In contrast to ABCG4, which apparently prevents megakaryocytes from proliferation, ABCB6 more likely modulates survival of megakaryocyte progenitors during oxidative stress due to its role in the mitochondrial porphyrin transport [57]. In addition, bone marrow ABCB6 deficiency was associated with accelerated atherosclerosis in mice [57]. Apart from these new insights into specific ABC transporter functions in atherogenesis and atherosclerosis-linked processes, it was already known that various ABC transporters are abundantly expressed in stem and progenitor cells of different origins [54]. It is therefore not surprising that knock out of those proteins may hamper stem cell homeostasis and differentiation [58].

### 3.3. ABCC4

Unlike the ABC transporters mentioned above, ABCC4 does not alter platelet counts, but rather affects signal transduction pathways known to control platelet activity. ABCC4 has been shown to transport cyclic nucleotides, such as cyclic adenosine monophosphate (cAMP) and cyclic guanosine monophosphate (cGMP), thus regulating intracellular cyclic nucleotide signaling [59,60]. In platelets, cAMP-dependent protein kinase (PKA) signaling modifies cytoskeletal reorganization, integrin activation, Ca^2+^ mobilization and granule secretion. Elevation of intracellular cAMP, which induces the activation of PKA, results in the inhibition of platelet function. According to its role as a cyclic nucleotide transporter, ABCC4 appears to be directly involved in platelet activation. First, it was shown that ABCC4 was highly expressed in platelets, but mainly localized to the membranes of dense (δ) granules and only to a lesser extent to the cell membrane [61]. Moreover, cGMP transport co-distributed with ABCC4 detection in subcellular platelet analyses in this study. Later on, studies on *ABCC4*^−/−^ platelets from transgenic mice substantiated the role of the transporter in homeostasis and platelet activation [62,63]. In these studies, it was shown that ABCC4 redistributed cAMP from the cytosol to dense granules, thereby reducing its availability in the cytosol and its ability to activate PKA. In line with these results, in ABCC4-deficient mice, a delayed arterial thrombosis, longer bleeding time and decreased collagen-dependent thrombus formation under flow conditions were observed [63]. Pharmacological experiments on *ABCC4*^−/−^ platelets with regard to PKA and adenylate cyclase (AC) activity, as well as ADP-dependent signaling to nucleotide receptors P2Y_12_ and P2Y_1_ revealed that (i) the kinetic response of PKA was significantly increased in *ABCC4*^−/−^ platelets and (ii) platelet activation specifically depends on P2Y signaling. These findings might be explained by defective cAMP and ADP storage as a result of ABCC4 depletion, ultimately leading to impaired platelet functions [62]. In summary, these findings might build a basis for further pharmacological approaches in the treatment of atherosclerosis-induced CVD [63,64].

## 4. ABC Transporters in the Regulation of Endothelial Function and Cardiovascular Homeostasis

Endothelial dysfunction is an important early event in the process of atherosclerosis and is involved in the pathogenesis of atherothrombotic complications. Apart from ABC transporters directly involved in lipid homeostasis and platelet functions, further ABC transporters, in particular of the ABCC family, have been implicated in endothelial homeostasis, the regulation of vascular tone and systemic blood pressure, as well as cardiomyocyte physiology.

### 4.1. ABCC1 and ABCC4

ABCC1 (multidrug resistance-associated protein 1 (MRP1)) was first discovered in cancer cells, where it contributes to the phenomenon of multidrug resistance, but has later on also been detected in a variety of organs and tissues, such as vascular endothelial cells, kidneys, lungs, spleen and adrenal glands [65]. It is involved in the extrusion and compartmental restriction of various hydrophobic xenobiotics and drugs, as well as in transport of glucuronide, sulfate and glutathione conjugates. Apart from this, ABCC1 has been shown to play a role in the regulation of vascular endothelial homeostasis and blood pressure by inducing glutathione efflux from vascular endothelial cells [66,67]. Interestingly, induction of pro-atherogenic oscillatory shear stress enhanced glutathione efflux in an ABCC1-dependent fashion in human endothelial cells in vitro. Moreover, hypertension-associated endothelial dysfunction was ameliorated in ABCC1-deficient mice in vivo. In addition, ABCC1-deficient mice were protected from angiotensin II-induced blood pressure elevation, vascular oxidative stress and endothelial glutathione depletion. These findings point to an important role of ABCC1 in vascular homeostasis and blood pressure regulation. In a subsequent study, ABBC1 was also directly implicated in the process of atherogenesis [68]. In this study, the ABCC1 inhibitor MK571 significantly improved endothelial dysfunction and reduced atherosclerotic lesion formation in the apolipoprotein E (ApoE) knockout mouse model of atherosclerosis. Taken together, ABCC1 might contribute to the onset and progression of CVD, and ABCC1 inhibition may represent a novel strategy to prevent hypertension, endothelial dysfunction and atherosclerotic vascular disease in high-risk cardiovascular patients.

As described above, ABCC4 as a transporter of cyclic nucleotides, mostly cAMP, regulates cyclic nucleotide homeostasis and thereby influences several cardiovascular processes. Besides being highly expressed in platelets, ABCC4 is found expressed in coronary [69] and pulmonary artery smooth muscle cells (SMCs) [60], as well as in atrial [70] and ventricular cardiomyocytes [71]. ABCC4 may therefore regulate cyclic nucleotide-dependent signaling in vascular SMC and cardiomyocytes and by this means affect vascular tone, SMC proliferation and cardiomyocyte pathophysiology. In this regard, ABCC4 expression was found to be elevated in SMCs during non-physiological/pathological conditions. Such elevated levels in ABCC4/MRP4 were observed in human coronary SMCs in vitro, in rat and mouse arteries after exposition to hypoxic conditions [69,71], as well as in patients with idiopathic pulmonary arterial hypertension [60]. Conversely, silencing of ABCC4 resulted in elevated cellular cyclic nucleotide levels, which in turn lead to activation of the corresponding downstream effectors protein kinase A (PKA) and protein kinase G (PKG). These effectors ultimately inhibited SMC proliferation both in vitro and in animal experiments after hypoxia induction in vivo. Furthermore, PKA and PKG have been attributed to the amelioration of pulmonary hypertension in animals.

In addition to its role in vascular tone and remodeling, ABCC4 may exert important effects in the myocardium via cyclic nucleotide efflux, in particular cellular cAMP depletion. Indeed, it is well known today that intracellular cAMP and extracellular adenosine levels are important factors when it comes to cardiomyocyte function and the behavior of fibroblasts in the cardiac syncytium. ABCC4 is responsible for cAMP efflux from cardiomyocytes into the extracellular space where it is stepwise metabolized to AMP and adenosine. Adenosine is then acting as a paracrine effector molecule on both cardiomyocytes and cardiac fibroblasts [72]. In cardiomyocytes, adenosine-mediated activation of adenosine receptor subtype 1 reduces cAMP levels through inhibition of adenylate cyclase activity, thereby reducing cardiomyocyte inotropy, hypertrophy and apoptosis. In contrast, extracellular adenosine induces cAMP production in cardiac fibroblasts via activation of adenosine receptor subtype 2 and consequently inhibits cardiac fibrosis [72].

Thus, while therapeutic ABCC4 inhibition might have the potential to reduce platelet-dependent ischemic events, it may also exert deleterious effects in the cardiac syncytium due to inhibition of cardioprotective ABCC4-dependent cAMP secretion [72]. Therefore, ABCC4 is not yet considered an applicable target for systemic treatment of CVD due to its various and not yet fully understood functions.

### 4.2. ABCC6

The role of ABCC6 in cardiovascular diseases is quite elusive. Even though it has been put into context with specific forms of abnormal tissue calcification syndromes like pseudoxanthoma elasticum [73], β-thalassemia [74] and generalized arterial calcification of infancy (GACI) [75], not much is known about the cardiovascular-specific actions of this ABC transporter. ABCC6 is broadly expressed in the liver, kidneys, intestine, retina and, to a much lesser extent, in other tissues, including the vasculature [76], but its substrate spectrum and tissue-specific functions remain enigmatic [77]. However, the elusive function of ABCC6 seems to be related to abnormal calcification of specific tissues, which are mostly the skin, kidneys, tendons and the cardiovascular system. Calcification is a prevalent feature of aging and also associated with atherosclerosis and coronary artery disease [78,79]. While it was long believed that abnormal tissue calcification results mainly from local, passive precipitation of calcium and phosphate, recent studies have described it as a complex process that involves osteoblastic re-differentiation of smooth muscle cells, pericytes and myofibroblasts [80]. Considering the inheritable calcification syndromes resulting from dysfunctional ABCC6, this transporter is now viewed as a key player in ectopic mineralization pathologies, but it is unclear how ABCC6 contributes to this process. Nonetheless, the latest studies indicate that loss of *ABCC6* alters gene expression profiles in a way that mineralization-promoting genes (tissue-nonspecific alkaline phosphatase (*TNAP*), bone morphogenetic protein (*BMP*)) are upregulated while the expression of genes with the opposite effect (ecto-5′-nucleotidase (*NT5E*), *Fetuin A* and *Osteopontin*) is decreased both in vitro [81] and in vivo [82]. Moreover, recent in silico studies have suggested new substrate candidates for ABCC6 that hopefully will be evaluated experimentally in vitro and in vivo in the near future [83].

### 4.3. ABCC9

ABCC9, also known as sulfonylurea receptor 2 (SUR2), is another type of ABC transporter and a component of the ATP-sensitive potassium (K_ATP_) channel that is responsible for ATP-dependent inward K^+^ transport. K_ATP_ channels are always comprised heterogeneously as combinations of the pore-forming subunits inward rectifier potassium channel 6.2 (Kir6.2) or 6.1 (Kir6.1) with one of the sulfonylurea receptors SUR1 (ABCC8) or SUR2 (ABCC9). The two main isoforms of ABCC9/SUR2 are SUR2A and SUR2B, which are abundantly, but heterogeneously expressed in cardiomyocytes, skeletal muscle and SMCs of the vasculature of different organs [84]. K_ATP_ channels are mainly responsible for providing the dominant resting K^+^ and act as metabolic sensors for stress and/or hypoxia. Mutations in ABCC9 have already been associated with CVDs. For example, certain mutations in exon 38 of ABCC9, encoding the C-terminal domain of SUR2A, have been identified to result in idiopathic dilated cardiomyopathy. In vitro analyses of those mutated ABCC9 transport molecules also revealed a delayed metabolic cycle, leading to slower potassium transport rates and response times. Especially under metabolic stress conditions, post-hydrolic ADP binding is increased, leading to impaired K^+^ transport and thereby distorting the electric coupling of cardiomyocytes. This phenomenon reduces cardiac stress adaptation and results in myocardial damage [85]. These data are in agreement with those from further studies suggesting a role of K_ATP_ channels in preventing cell damage from intracellular Ca^2+^ overload, cardiac stress adaption and preconditioning-induced protection of myocardial energetics. Impaired K^+^-channel functions will therefore lead to predispositions for myocardial damage [85], survival disadvantages under stress [86] and loss of cardioprotective responses to ischemic preconditioning [87]. In addition, experimental evidence from *SUR2*^−/−^ knockout mice points to an important role of ABCC9 in the regulation of vascular tone (Table 1). In those animals, elevated resting blood pressure was observed, as well as cases of sudden death due to elevated ST segments and coronary artery vasospasm [88].

## 5. ABC Transporters in the Pharmacological Treatment of CVD

Several ABC transporters are known to contribute to the pharmacokinetics and disposition of drugs frequently used to treat CVD and CVD-related complications and may thus affect the efficacy and safety of these therapeutic interventions. In addition, genetically-determined transporter dysfunction or ABC transporter-associated drug interactions may lead to substantial changes in drug exposure and adverse effect profiles. Among the most relevant ABC transporters, ABCB1 (P-glycoprotein) has been demonstrated to influence absorption, distribution and elimination of a large number of drugs and to maintain the functional integrity of vital biological barriers [89,90]. Furthermore, increasing evidence points to a major role of two additional ABC-transporters, ABCG2 (breast cancer resistance protein (BCRP)) and ABCC2 (multidrug resistance-associated protein 2 (MRP2)), in the transport of numerous drugs and phase II metabolites, i.e., conjugates of organic anions with sulfate, glutathione or glucuronic acid [91]. ABCB1, ABCG2 and ABCC2, besides being known for their important role in multidrug resistance of cancer cells against chemotherapeutic agents, are all localized at biological interfaces between the organism and its environment (e.g., the apical membrane of enterocytes, the canicular membrane of hepatocytes). Besides their localization on interfaces with the environment, they also control metabolic exchange between the blood and important body compartments (blood-brain barrier, testis, placenta), thereby emphasizing their impact on drug pharmacokinetics and distribution [90]. In this passage, we will very concisely review the role of ABC transporters in the pharmacokinetics of selected drug classes frequently used to treat CVD pathologies.

### 5.1. Interaction of Statins with ABC Transporters

Statins or 3-hydroxy-3-methyl-glutaryl-coenzyme A (HMG-CoA) reductase inhibitors are important lipid altering agents that are commonly used for the treatment of hypercholesterolemia, as well as for primary and secondary prevention of coronary heart disease and stroke [92,93]. On the pathophysiological level, beneficial mechanisms in atherosclerotic vascular disease include plaque regression and stabilization, reversal of endothelial dysfunction and decreased thrombogenicity. However, the absolute clinical benefits of statin therapy depends on the achieved reduction in LDL cholesterol [94,95]. Most statins are pharmacokinetically characterized by a low oral bioavailability due to substantial hepatic uptake (mediated in relevant parts by uptake transporters, such as organic anion-transporting polypeptide 1B1 (OATP1B1)) and subsequent pre-systemic elimination or inactivation, mainly via cytochrome P450 oxidases, such as CYP3A4 (atorvastatin, lovastatin, simvastatin) or CYP2C9 (fluvastatin) [96]. Although statins have a favorable risk-to-benefit ratio and are generally well tolerated by most CVD patients, they may induce myotoxicity, especially in patients receiving high-dose treatment [97]. In this context, drug-drug interactions can increase systemic statin exposure and considerably increase the risk of myotoxicity in affected patients. As several ABC transporters have been especially implicated in the intestinal elimination of statins, drug-drug interactions that involve inhibition of ABC transporter-mediated statin efflux may increase the exposure to and hamper the tolerability and safety of these key anti-atherosclerotic agents.

The particular ABC transporters ABCB1, ABCG2, as well as ABCC2 have been shown to be capable statin efflux pumps [96,98]. Depending on the (partly overlapping) substrate specificity of the respective ABC transporter, affinity for certain statins differs. For instance, both ABCB1 and ABCG2 efficiently transport atorvastatin, lovastatin and simvastatin [99], whereas ABCG2 and to a minor extent also ABCC2 may be of importance for the pharmacokinetics of rosuvastatin [100]. There are already several genetic polymorphisms known for all three members of the ABC transporter family, which have been shown to affect statin pharmacokinetics and tolerability, even though their respective carrier frequency is often unknown [101]. The ABCG2 polymorphism C421A for example is associated with lower protein expression in vitro [102,103] and has been shown to increase exposure to atorvastatin and rosuvastatin in human individuals by 72% and 100%, respectively [99]. Moreover, statins may also modulate ABC transporter expression, e.g., the expression of ABCC2, in relevant tissues and by this means affect the pharmacokinetics of co-administered drugs [104,105]. Keeping in mind that CVD patients often receive polymedications, it is conceivable that these individuals are at risk of developing harmful ABC transporter-related drug-drug interactions. Thus, further basic and clinical research is needed to improve the efficacy and safety of statin use in CVD patients.

### 5.2. Interaction of Angiotensin Receptor Blockers with ABC Transporters

Angiotensin receptor blockers (ARBs, angiotensin II subtype 1 receptor antagonists) are increasingly prescribed drugs commonly used to treat patients with hypertension and heart failure. Moreover, they are integral components of the novel combination angiotensin receptor-neprilysin inhibitor (ARNi) drug class that has been approved recently in Europe and the U.S. for the treatment of heart failure. ARBs have long been viewed as agents with low interaction potential as they do not relevantly interact with cytochrome p450 oxidases [106]. Nonetheless, it was recently discovered that ARBs, such as telmisartan or the prodrug candesartan cilexetil, interact with ABC transporters like ABCB1, ABCC2 and ABCG2 [107,108]. In this context, telmisartan was identified to be the ARB with the highest potential for ABC transporter interaction. It is a potent ABCB1 and moderate inhibitor of ABCC2 and ABCG2 [108,109]. Even though a clinical study on healthy volunteers administering telmisartan and the prototypical ABCB1 substrate digoxin showed significant increase in the AUC and maximum serum concentration (C_max_) of digoxin, the clinical relevance of this finding remains elusive [109,110], mostly because only intestinal telmisartan concentrations might be high enough for effective inhibition of ABCB1 [110]. Nevertheless, since ABCB1 has a broad substrate range and is known to transport a vast number of drugs ranging from anticancer drugs to HIV protease inhibitors, antiemetics, cardiac glycosides, calcium channel blockers, immunosuppressive agents and antibiotics [111], the interaction potential should not be underestimated. At least with regard to CVD treatment, the simultaneous use of statins and angiotensin receptor blocker may carry some risk of drug-drug interactions as indicated by recent clinical trials involving telmisartan and rosuvastatin [112].

### 5.3. Interaction of Aspirin and Clopidogrel with ABC Transporters 

Aspirin and clopidogrel (in either single or combined use) are the most important antiplatelet drugs that are prescribed to prevent acute ischemic events in individuals who are at high cardiovascular risk, including patients with a history of myocardial infarction, stroke and peripheral artery disease [113,114]. Aspirin is a potent cyclooxygenase 1 (COX-1) inhibitor and especially prevents the COX-1-dependent thromboxane A_2_ production in platelets [115]. It thereby significantly decreases platelet activation and aggregation. Due to its clinical effectiveness and low costs, aspirin has become the “gold standard” for the prevention of atherothrombotic complications in CVD patients [114]. Nonetheless, prevalent prescription of aspirin for secondary prevention in CVD has revealed that some patients do not respond appropriately and develop some kind of resistance towards the antiplatelet function of aspirin, a phenomenon also known as “aspirin resistance” [116]. Especially after coronary artery bypass grafting (CABG), a subgroup of patients does not adequately respond to aspirin, a clinical observation that was first attributed to the massively increased platelet turnover following the almost complete platelet depletion during the surgical procedure [117]. It was later found that not the elevated platelet turnover alone, but a significant upregulation of ABCC4 might be responsible for the decreased aspirin response in CABG patients, since aspirin can be extruded from platelets through ABCC4/MRP4-mediated efflux [118]. Even low-dose aspirin treatment alone has been shown to induce ABCC4 upregulation in human megakaryocytes and platelets in a peroxisome proliferator-activated receptor-α (PPARα)-dependent fashion. Hence, this mechanism might contribute to the phenomenon of aspirin resistance [119].

Clopidogrel on the other hand is a thienopyridine antiplatelet agent that inhibits ADP-mediated platelet activation and aggregation by selectively and irreversibly blocking platelet purinergic P2Y12 receptors. As an inactive prodrug, clopidogrel needs hepatic biotransformation catalyzed by cytochrome P450 oxidases, i.e., CYP1A2 [120]. In previous studies, a considerable inter-individual variability in the response to clopidogrel treatment has been described that may in part depend on the availability of active CYP1A2. In addition, numerous studies have demonstrated that the ABC transporter ABCB1 limits intestinal clopidogrel uptake, thereby reducing its oral bioavailability [121,122]. In this regard, pharmacogenetic analyses have revealed that ABCB1 variants, e.g., the C3435T polymorphism, reduce the clinical efficacy of clopidogrel, which clearly demonstrates the importance of ABCB1 in clinical clopidogrel use [120].

## 6. Conclusions and Perspectives

Although ABC transporters have been extensively investigated in diseases, such as cancer and cancer multidrug resistance, their importance in the pathogenesis of CVD is just beginning to be elucidated. For example, ABCA1 has been identified as an ABC transporter with a crucial role in HDL formation, as well as in the cellular efflux of cholesterol and lipids from vascular endothelial cells and macrophages. This relation is underscored by the finding that ABCA1 mutations are highly prevalent in individuals with low HDL levels. Thus, pharmacological manipulation of ABCA1 activity may represent an innovative strategy to improve the lipid profile and vascular function of CVD patients in the future. Moreover, increasing knowledge regarding the precise role of ABC transporters in CVD initiation and progression may pave the way toward the development of novel pharmacological agents capable of ameliorating endothelial dysfunction and atherosclerotic vascular disease in high-risk cardiovascular patients. In addition, ABC transporters have an important role in the pharmacokinetics and disposition of drugs frequently prescribed in CVD patients and are therefore related to the efficacy and safety of these therapeutic interventions. For instance, the pharmacokinetics of statins, a drug class heavily used for primary and secondary prevention of, i.e., coronary heart disease, are significantly influenced by ABC transporters, such as ABCB1 and ABCG2. Hence, CVD patients receiving multiple agents for cardiovascular risk reduction are at risk of developing harmful ABC transporter-related drug-drug interactions that may foster statin-induced side effects, such as myotoxicity. In this context, genetic variability in ABC transporter genes may contribute to interindividual variability in the cholesterol-lowering effect of statins, but also in the anti-platelet effects of clopidogrel. Thus, future efforts are urgently needed to improve our knowledge regarding the impact of ABC transporters in cardiovascular pharmacology to maximize the efficacy and safety of CVD therapeutics.

## Figures and Tables

**Table 1:** Adenosine triphosphate (ATP)-binding cassette (ABC) transporters involved in pathophysiology of cardiovascular diseases (CVD).

ABC Transporter	Function	Relevance for CVD
ABCA1	HDL formation by loading phospholipids on ApoA-1	Involved in lipid and cholesterol clearance [17,18,19]; involved in hematopoiesis [26,27]
ABCB4	Hepatic regulation of phosphatidylcholine secretion into bile; scavenge of atherogenic particles in Kupffer cells and macrophages	Influences cholesterol levels and foam cell formation [50]
ABCB6	Involved in mitochondrial porphyrin transport in hematopoietic stem cells	Influences platelet counts and platelet activity [57]
ABCC1	Involved in export of glutathione disulfide from vascular endothelial cells	Enhances hypertension, endothelial dysfunction and atherosclerotic lesion formation [66,67,68]
ABCC4	Nucleotide transporter controlling intracellular cAMP/cGMP signaling	Mediates vascular tone [60] and platelet activation [62,63]
ABCC6	Mode of action still unknown	Dysfunction increases risk for vascular calcification and myocardial infarction [78,79,80]
ABCC9	As K^+^-channel responsible for providing the dominant resting K^+^ conductance	Idiopathic dilated cardiomyopathy [85]; elevated resting blood pressure; carotid artery vasospasm [88]
ABCG1	Cellular oxysterol efflux to mature HDL; reverse cholesterol transfer pathway	Cholesterol and phospholipid deposition [37]; accelerated foam cell formation and risk for artery stenosis [38]; involved in hematopoiesis [26]
ABCG4	Sensing and efflux of membranous cholesterol to HDL	Loss of function leads to increased proliferation of megakaryocyte progenitors and platelet counts [51]
ABCG5/G8	Hepatic efflux of cholesterol and plant sterols into the bile	Regulates serum cholesterol levels [18,42,43,44]

HDL, High-density lipoprotein; ApoA-1, apolipoprotein A1; cAMP/cGMP, cyclic adenosine/guanosine monophosphate.
